# Supplementary material for: From sequence to enzyme mechanism using multi-label machine learning
Source: BMC Bioinformatics. 2014 May 19;15:150. doi: 10.1186/1471-2105-15-150 (PMC4229970; doi:10.1186/1471-2105-15-150)
Supplement: Additional file 2 — Java code of ml2db. Additional file ml2db_code.tar.gz contains the Java source code to run the multi-label machine learning experiments and save the results to database. The code’s Javadoc is included. [file 1471-2105-15-150-S2.zip › additional file 2/ml2db/ml2db/doc/test/mulan/mulanarff/StatisticsTest.html]

StatisticsTest


---


|  |  |  |
| --- | --- | --- |
| **Overview** \| **Package** \| **Class** \| **Use** \| **Tree** \| **Deprecated** \| **Index** \| **Help** | |  |
| **PREV CLASS**   NEXT CLASS | **FRAMES**    **NO FRAMES**     **All Classes** |  |
| SUMMARY: NESTED \| FIELD \| CONSTR \| METHOD | DETAIL: FIELD \| CONSTR \| METHOD |  |


---


## test.mulan.mulanarff Class StatisticsTest

```
java.lang.Object
  test.mulan.mulanarff.StatisticsTest
```

---

``` public class StatisticsTest extends java.lang.Object ```

Class

**Version:**
:   12 Apr 2012

**Author:**
:   Luna De Ferrari luna.deferrari@ed.ac.uk or
    luna.deferrari@st-andrews.ac.uk

---

**Constructor Summary**

`StatisticsTest()`


| **Method Summary** | |
| --- | --- |
| `static Statistics` | `getStats(IdMultiLabelInstances data)` |
| `static Statistics` | `getStatsTestArff()` |
| `static Statistics` | `getStatsTrainArff()` |
| `void` | `testGetNumInstances()`             Test method for `Statistics.getNumInstances()`. |

**Methods inherited from class java.lang.Object**

`equals, getClass, hashCode, notify, notifyAll, toString, wait, wait, wait`

**Constructor Detail**

### StatisticsTest

```
public StatisticsTest()
```


**Method Detail**

### testGetNumInstances

```
public void testGetNumInstances()
```

:   Test method for
    `Statistics.getNumInstances()`.

---


### getStats

```
public static Statistics getStats(IdMultiLabelInstances data)
```

---


### getStatsTestArff

```
public static Statistics getStatsTestArff()
```

---


### getStatsTrainArff

```
public static Statistics getStatsTrainArff()
```


---


|  |  |  |
| --- | --- | --- |
| **Overview** \| **Package** \| **Class** \| **Use** \| **Tree** \| **Deprecated** \| **Index** \| **Help** | |  |
| **PREV CLASS**   NEXT CLASS | **FRAMES**    **NO FRAMES**     **All Classes** |  |
| SUMMARY: NESTED \| FIELD \| CONSTR \| METHOD | DETAIL: FIELD \| CONSTR \| METHOD |  |


---
